# A single-arm pilot study of a brief cognitive-behavioral therapy for insomnia intervention among Japanese occupational therapy and physical therapy university students with sleep disturbances

**DOI:** 10.3389/frsle.2024.1397311

**Published:** 2024-10-14

**Authors:** Yuki Kawakatsu, Miki Takahata, Shinji Satake, Toshiaki Sato, Aaron Eakman

**Affiliations:** ^1^Department of Occupational Therapy, Yamagata Prefectural University of Health Sciences, Yamagata, Japan; ^2^Department of Occupational Therapy, Colorado State University, Fort Collins, CO, United States

**Keywords:** sleep quality, mental health, patient-reported outcome, cultural adaptation, occupational therapist, meaningful activity

## Abstract

**Objective:**

To evaluate the feasibility of the Sleep Health through University Student Habits (SHUSH) program, a brief sleep improvement intervention based upon principles of cognitive-behavioral therapy for insomnia (CBT-I) developed for Japanese university students.

**Methods:**

Pretest-posttest, single-arm pilot study design with 3-month follow up was used to evaluate the feasibility of SHUSH. We developed then offered a 90-min sleep education class based upon the two-process model of sleep regulation, sleep restriction, stimulus control, and sleep hygiene. We recommended individualized sleep prescriptions (e.g., prescribed time to bed and prescribed time out of bed) from 11 days of daily sleep diary data. We then offered 15 min of individualized follow-up meetings each week for three consecutive weeks in person or online in which we supported adherence to sleep prescriptions. Fifteen university students with self-reported sleep disturbance (Insomnia Severity Index; ISI score ≥ 9) completed the SHUSH program. Participants were on average age 19.7 years old; nine were women, and they were students in occupational therapy and physical therapy. We assessed validated Japanese versions of sleep-related (e.g., ISI, Sleep hygiene practice scale; SHPS), mental health-related (e.g., Generalized Anxiety Disorder Screener) patient-reported outcomes (PROs) and daily sleep diary variables (e.g., sleep onset latency and sleep efficiency).

**Results:**

Comparing baseline and posttest data, a statistically significant difference was observed in insomnia symptom severity, daytime sleepiness, sleep hygiene practices, eveningness to morningness, anxiety, depression, sleep diary improvements (e.g., sleep onset latency, total time in bed, and sleep efficiency). Comparing posttest and 3-mo follow up data (*n* = 10), a statistically significant difference wasn't observed for most PRO effects. However, there was a statistically significant difference in ineffective sleep behaviors (i.e., SHPS-J). We did observe a rebound effect for some SHPS-J items.

**Conclusion:**

SHUSH was developed as a brief cognitive-behavioral intervention for insomnia. SHUSH participants showed improvements on sleep-related and mental health-related PROs after 4 weeks of intervention. At 3 months follow-up sleep quality and mental health gains were maintained. SHUSH was a feasible program. Randomized controlled trials are needed to test treatment efficacy on sleep related and mental health related PROs in the future.

## 1 Introduction

The prevalence of insomnia in university students is 18.4%, considerably higher than 7.4% in the general population (Jiang et al., 2015) making insomnia in university students a problem with great social impact. Insomnia in students is associated with low mental health (Taylor et al., 2013) impaired daytime functioning [e.g., late arrival and dozing in class (Kayaba et al., 2020) and degraded academic performance (Vedaa et al., 2019)]. Promoting sufficient sleep (e.g., duration and timing) can foster sleep health and wellbeing (Ness and Saksvik-Lehouillier, 2018; Buysse, 2014). Interventions capable of reducing insomnia ultimately foster wellbeing and engagement in meaningful activities (Eakman et al., 2022).

Best practice in the treatment of chronic insomnia is cognitive-behavioral therapy for insomnia (CBT-I). A recent meta-analysis of CBT-I for university students (Takano et al., 2022) has shown its effectiveness. CBT-I for university students can be delivered face-to-face and/or virtually via telehealth (Takano et al., 2022; Taylor et al., 2014; Trockel et al., 2011) though face-to-face delivery may offer greater benefit (Lancee et al., 2016). In general, CBT-I is delivered by a behavioral health professional with advanced training in the intervention that includes 6–12 weekly sessions (Manber and Carney, 2015). This approach, though effective, may also be a burdensome commitment for those with insomnia. In recent years, brief CBT-I, which may be a combination of one or two one-hour sessions followed by short weekly individual sessions has been developed. The use of brief CBT-I has been expanding, especially for older adults in countries around the world (Buysse et al., 2011). In Japan, brief CBT-I for older adults performed by nurses demonstrated reductions in sleep onset latency and depression (Tanaka et al., 2019). Multicomponent CBT-I is now being effectively provided by varied professions with advanced training such as nurses (Tanaka et al., 2019) and occupational therapists (Eakman et al., 2022; Muench et al., 2022; Green and Hicks, 2015).

University students in Japan have a unique constellation of sleep concerns for which a tailored CBT-I intervention is necessary. In Japan, the prevalence of insufficient sleep syndrome (ISS) is 11%, and is highly prevalent in university students and those employed full-time (Morita et al., 2015). ISS, also known as “chronic insufficient sleep” is defined by a short sleep duration (e.g., < 6 h) and often co-occurs with frequent napping. Napping, however, is known to decrease the drive for sleep at night and may delay a person's sleep phase (Borbély et al., 2016; Chattu et al., 2018). Interventions addressing napping behaviors and their effects on sleep can be a key element of CBT-I for Japanese university students.

As well, the use of smartphones, especially in bed can contribute to ISS and poor sleep. A recent study of physical therapy and medical nursing students aged 18–30 years (Kawada et al., 2017) found smartphone use in bed at night was 3.4 h on average (range 0.5–6 h) and 85% of the sample kept their mobile phones in or near their beds. Kawada et al. (2017) mentioned that the reason of bringing their mobile phones near or in bed was due to feeling a need to respond quickly to their colleagues and friends. These habits can contribute to a delayed sleep phase. Multicomponent CBT-I including sleep hygiene education can tackle poor sleep hygiene practices, including in-bed smartphone use in bed.

As described above, the high prevalence of insomnia among Japanese university students is due to their poor sleep habits, such as napping and using smartphones in bed. Multicomponent CBT-I is the method of choice as it can modify cognitive and behavioral factors contributing to sleep problems.

A recent study found over 40% of Japanese university students had a sleep duration of < 6 h (Tanaka et al., 2019). It is assumed that Japanese university students fall asleep late due to a lifestyle that delays the sleep phase (such as napping and using smartphones in bed), resulting in not getting enough sleep. In order to improve the sleep of Japanese university students, it is necessary to establish a regular rhythm of falling asleep and waking up with support for time management, and to improve lifestyles that hinder good quality sleep. For that purpose, multi-component CBT-I is required given it promotes behaviors which can improve sleep regularity, quality, and quantity.

The purpose of this study was to develop and test the Sleep Health through University Student Habits (SHUSH) program, a brief CBT-I intervention. SHUSH was based upon the Restoring Effective Sleep Tranquility (REST) intervention that was effective in reducing insomnia and promoting mental health in post 9/11 Veterans (Eakman et al., 2022, 2017). The following considerations were made in the development of SHUSH: (1) Incorporating elements of multi-component CBT-I (e.g., sleep restriction therapy, stimulus control therapy, and sleep hygiene education), (2) Combining one face-to-face sleep education session with three individualized weekly follow-up sessions, (3) Creating the intervention to best suit the nature of Japanese university students' sleep challenges and lifestyles.

## 2 Method

### 2.1 Participants and procedure

**Figure 1:**
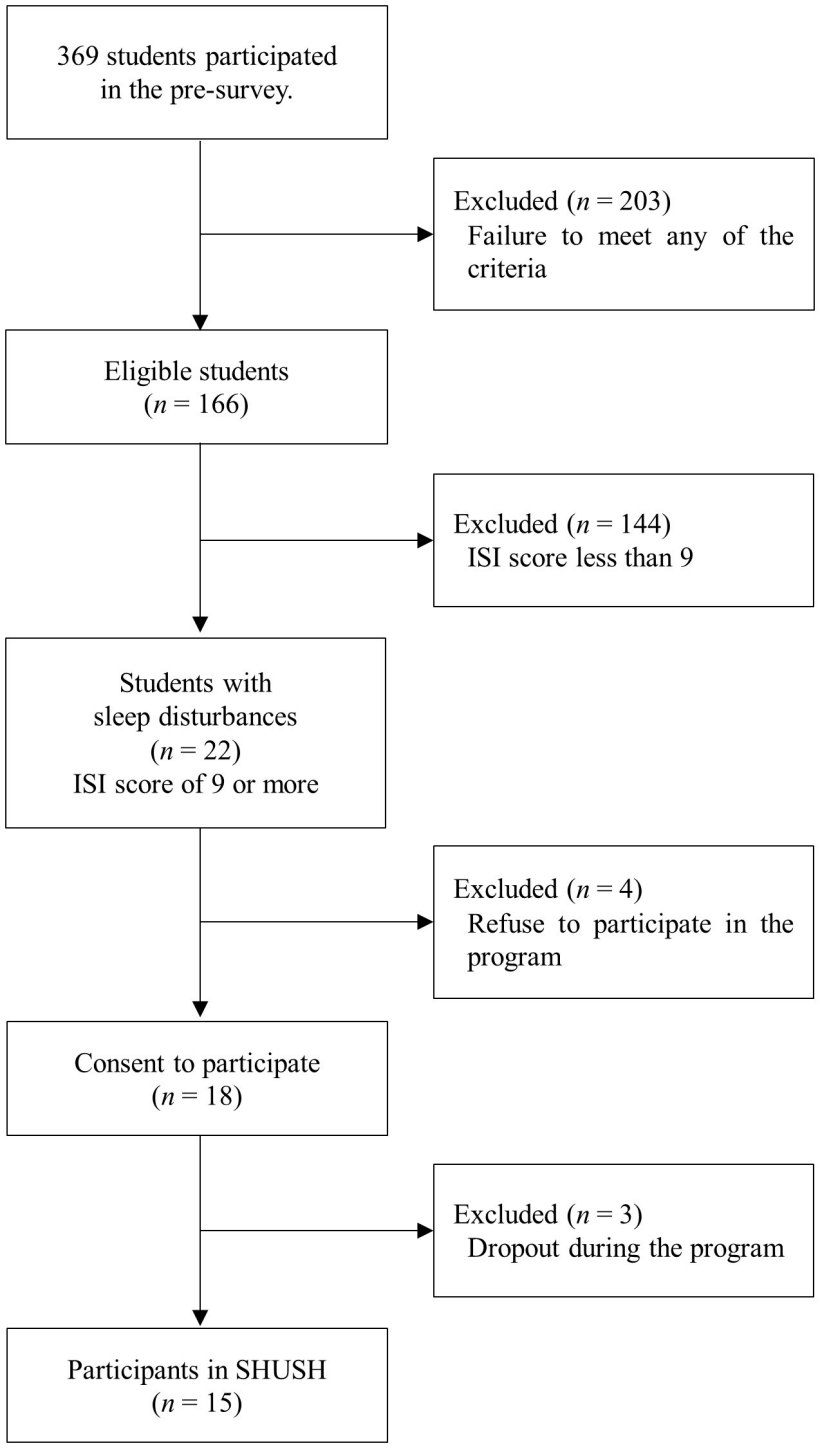
Flow chart of participant selection.

Inclusion criteria were as follows: prefectural health sciences university students including undergraduate nursing, occupational therapy, and physical therapy students; self-reported sleep disturbance (Insomnia Severity Index-Japanese version; ISI-J score ≥ 9); commitment to attend 1.5 h of sleep education; commitment to complete a daily sleep diary for 4 weeks and attend three 1:1 follow-up sessions. Exclusion criteria were mental illness under treatment; significant restless legs or obstructive sleep apnea symptoms; inability to attend both the 1.5-h sleep education class and three 15-min follow-up sessions. Exclusion criteria were assessed by using self-report questionnaire. Participants were undergraduate nursing, occupational therapy, or physical therapy students recruited from Yamagata Prefectural University of Health Sciences in Japan. A total of 369 students participated in a survey to select participants who met the inclusion criteria. The recruitment surveys were administered in-person by Eakman et al. We obtained written consent from 18 students. Three were excluded because they did not participate in the initial sleep education class. Thus, 15 students ultimately participated in the SHUSH program (Figure 1). Throughout the term of the recruitment survey and SHUSH program, this institution had no COVID-19 cases.

Survey data were collected October 2021. The SHUSH intervention was implemented November to December 2021 and three-month follow-up data were collected March 2022. This study was conducted with the approval of the Yamagata Prefectural University of Health Sciences institutional review board (# 2108-14).

### 2.2 Sleep Health through University Student Habits program

SHUSH is a four-week sleep health promotion intervention, based upon principles of multicomponent CBT-I including sleep restriction, stimulus control, and sleep hygiene. This program was delivered by Japanese occupational therapists (Yuki Kawakatsu and Miki Takahata) who were supervised by a psychologist (Shinji Satake) and occupational therapist (Aaron M. Eakman) with expertise in CBT-I. To prepare the occupational therapy interveners, the team reviewed the REST protocol, a multicomponent CBT-I program for treating chronic insomnia in post 9/11 Veterans (Eakman et al., 2022, 2017). The SHUSH OT interveners were educated on sleep physiology (e.g., two-process model of sleep regulation), as well the principles and practice of multicomponent CBT-I. Cognitive (limit worry about sleep and behavioral mechanisms, e.g., waking at the same time each day, napping) that can promote effective sleep were discussed as part of a brief translation and cultural adaptation of the REST protocol into Japanese.

From this process we developed SHUSH, a 4-week sleep health promotion intervention for university students in Japan. In W 1, we offered 90 min of sleep education in a university lecture hall. The sleep education consisted of a 60-min lecture and a 30-min individual session to develop individualized sleep prescriptions for each participant. The lecture was developed based on REST by Japanese OT interventionists under the guidance of the REST developer (Aaron M. Eakman) to make it easier for Japanese university students to understand. Lecture content included a review of sleep physiology addressing the two-process model of sleep regulation (i.e., circadian rhythm and homeostatic sleep-driver system synchrony) (Borbély et al., 2016). We also explained sleep-promoting behaviors and clarified their role in minimizing sleep disturbances (see Table 1).

**Table 1:** Techniques for improving sleep.

1. Go to bed and wake up at the same time to maintain circadian rhythm (use an alarm clock)
2. Get out of bed as soon as you wake up in the morning (soak in the sun)
3. Be active during the day to increase sleep drive.
4. Do not take a nap to increase sleep drive.
5. Get out of bed when you can't sleep and go back to bed when you get sleepy.

**Figure 2:**
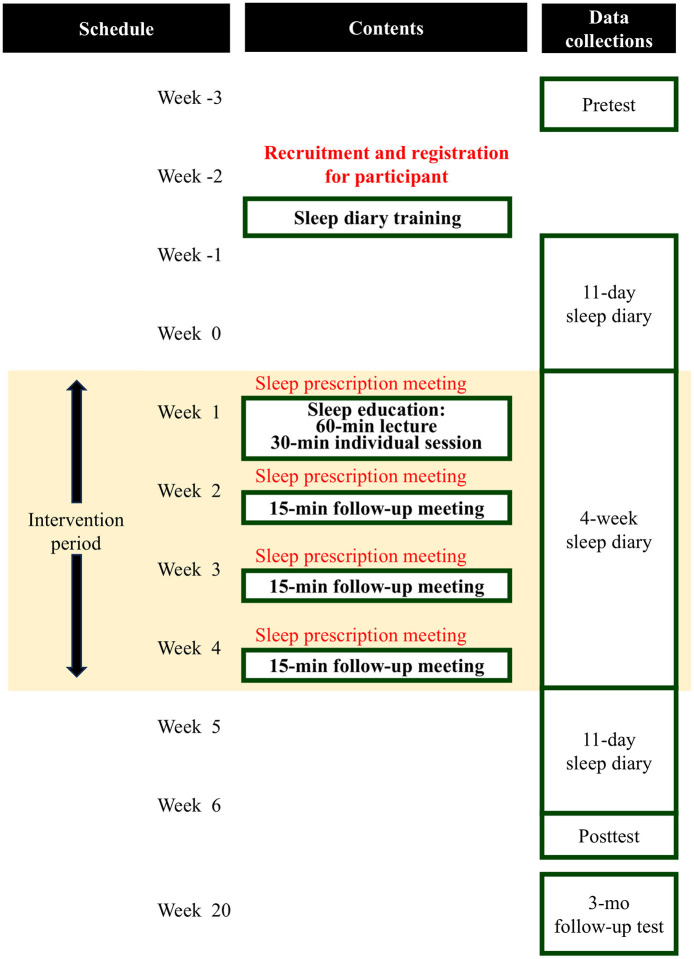
Study design: Sleep Health through University Student Habits program.

Then, we recommended individualized sleep prescriptions to participants as the final aspect of the 90-min sleep education. Individualized sleep prescriptions were based upon 11 days of consensus sleep diary (Carney et al., 2012) data completed 2 days prior to the 90-min sleep education. We set individuals' prescribed time to bed and out of bed in consideration of the student's total sleep time, allowing no < 6 h of sleep opportunity (Troxel et al., 2012). We also attempted to accommodate for participants' daily routine (e.g., class start time, part-time job finish time). In Weeks 2, 3, and 4 we offered 15 min of individualized follow-up meetings each week in person or online. In these meetings, we provided feedback to promote understanding of and suggestions for adherence to sleep prescriptions. If participants were having difficulty with adhering (e.g., going to bed later than their prescribed time to bed), we explored their reasons and offered solutions for managing their time. As a general rule, we added 15 min to participants' total time in bed each week in the case that their sleep efficiency was at or above 90%. To support treatment fidelity, we discussed and agreed to sleep prescriptions prior to both the 90-min education session and the three 15-min follow-up sessions (Figure 2).

### 2.3 Instruments

We assessed demographic variables, sleep diary outcomes, and sleep-related, mental health-related, and participation-related patient-reported outcomes (PROs) at baseline and posttest. Baseline data were collected ~2 weeks prior to the start of the sleep education class, except for 11 days of sleep diary data collected immediately prior to the sleep education class. Posttest data were collected within 1 week following the final 15-min follow-up meeting and again 14 weeks later.

#### 2.3.1 Demographic variables

We included age in years, gender, year in school, daily time commuting to school, weekly hours spent working or volunteering and participating in clubs.

#### 2.3.2 Patient-reported outcomes (PROs)

##### 2.3.2.1 Sleep-related outcomes

We included the ISI-J (Munezawa, 2009), which assessed severity of sleep disturbances (cut- off point ≥ 9–11) (Fabbri et al., 2021), the Japanese version of the Pittsburgh Sleep Quality Index (PSQI-J) (Doi et al., 2000), which assessed sleep quality (cut- off point ≥ 5.5), the Japanese versions of the Epworth Sleepiness Scale (J-ESS) (Takegami et al., 2009), which assessed daytime sleepiness (cut- off point ≥ 11), the Japanese version of the Dysfunctional Beliefs and Attitudes about Sleep Scale (DBAS-J) (Munezawa et al., 2009) which assessed inaccurate sleep beliefs, the Japanese version of the Sleep Hygiene Practice Scale (SHPS-J) (Hara et al., 2021), which assessed factors which negatively impact sleep quality, the Japanese version of the Morningness-Eveningness Questionnaire (MEQ-J) (Ishihara et al., 1984), which assessed participants' chronotype preference (16–41 points = night type; 42–55 points = intermediate type; 56–86 points = morning type).

##### 2.3.2.2 Mental health-related outcomes

We included the Japanese version of the Generalized Anxiety Disorder-7 Screener (J-GAD-7) (Muramatsu et al., 2010; Muramatsu, 2014), which assessed generalized anxiety (0-4 points = normal; 5–9 points = mild; 10–14 points= moderate; 15–21 points = severe). The Japanese version of the Center for Epidemiologic Studies Depression Scale (J-CESD) (Shima et al., 1985), which assessed severity of depression symptoms (cut- off point ≥ 17).

##### 2.3.2.3 Participation-related outcomes

We included the Japanese version of the Engagement in Meaningful Activities Survey (EMAS-J) (Kawakatsu et al., 2022), which assessed experiences of positive meanings associated with day-to-day activities within persons' daily life.

#### 2.3.3 Sleep diary outcomes

The consensus sleep diary (Carney et al., 2012) was used in the present study. We translated the original English version into Japanese. Participants were instructed to make a record of their sleep each morning. Every week, they submitted their sleep diary as a pdf or photo to the two interveners. That information was used to generate sleep diary variables which were used to establish and modify sleep prescriptions. These data included sleep onset latency, nighttime awakening, wake after sleep onset, total time in bed, total sleep time, and sleep efficiency.

### 2.4 Study design

This was a single-arm pretest—posttest effectiveness study (Figure 2). Participants completed a survey including a demographic questionnaire, sleep-, mental health-, and participation-related PROs which were used to enroll students in SHUSH. All participants participated in a 30-min sleep diary training to learn how to accurately record sleep diary data. As mentioned above, we collected 11 days of sleep diary data immediately before the sleep education offered in Week 1. Participants maintained a daily sleep diary for the next few weeks for use in the follow-up meetings which were held Weeks 2, 3, and 4. Post-test sleep diary data were collected 11 days following participants' final follow-up meeting. Posttest sleep-, mental health-, and participation-related outcomes were collected immediately following the 11-day posttest sleep diary period. Participants again completed PROs at 3-mo follow-up about 14 weeks after completing the posttest PROs.

### 2.5 Data analysis

There were no missing data in PROs. Regarding the calculation of sleep diary outcomes, data for 9 days were used for the calculation of 2 participants at the baseline due to omissions in the sleep diaries. In the posttest, due to omission of sleep diaries and delays in the final follow-up schedule, 6 days of data for 1 participant, 7 days of data for 1 participant, 9-day data for 2 participants, and 10-day data for 4 participants was used for the calculation. Data analysis included descriptive statistics for baseline demographic variables. The normality of the sleep diary outcomes and PROs were examined using the Shapiro-Wilk test. Dependent *t*-tests were used for analysis of normally distributed data, while Wilcoxon rank sum tests were used for analysis of non-normally distributed data. In addition, effect sizes were also calculated as *r*. These were used to explore differences in PROs and sleep diary outcomes between baseline and posttest and posttest and 3-mo follow up with *p* < 0.05 set to indicate significant differences. In addition, for SHPS-J, paired *t*-tests or Wilcoxon rank sum tests of baseline to posttest and posttest to 3-mo follow-up were performed for each item. These tests were used to explore SHPS-J item-level changes associated with the SHUSH intervention.

## 3 Results

In this study, 15 students completed the SHUSH program (see Figure 1). Their mean (SD) age in years was 19.7 (1.5), range 18–22, and 9 (60.0%) were female. They were in 1st, 2nd, and 4th grade: 1st *n* = 8 (53.3%), 2nd *n* = 3 (20.0%), 4th *n* = 4 (26.7%). Students belonged to the department of physical therapy or occupational therapy: physical therapy *n* = 9 (60.0%), occupational therapy *n* = 6 (40.0%). Most students (*n* = 12, 80.0%) commuted < 30 min to school each day. Most students (*n* = 14, 93.3%) reported working or volunteering: 15 h a week *n* = 3 (20.0%), 20 h a week *n* = 4 (26.7%), 25 h a week *n* = 2 (13.3%), 30 h or more a week *n* = 2 (13.3%). A minority of students (*n* = 5, 35.7%) reported weekly involvement in a circle or club, requiring < 5 h a week.

We found very large improvements in sleep efficiency (*r* = 0.880, *p* = 0.000) and very large reductions in sleep onset latency (*r* = 0.851, *p* = 0.000) and total time in bed (*r* = 0.718, *p* = 0.002) when comparing baseline and posttest data SHUSH intervention data (see Table 2). Though these improvements were evident, students did not report appreciable gains in total sleep time which remained at ~6.5 h on average.

**Table 2:** Change in sleep diary indicators from baseline to posttest.

**Variable**	**Baseline, mean (SD)**	**Posttest, mean (SD)**	**Change from baseline to posttest (*****n*** = **15)**	
			**Effect size, *r***	***p*-value**
Sleep onset latency	56.04 (33.65)	25.45 (38.01)	0.851	0.000^†^
Nighttime awakenings	1.05 (0.88)	0.53 (0.61)	0.514	0.048^†^
Wake after sleep onset	12.49 (13.86)	6.76 (10.80)	0.334	0.216^†^
Total time in bed	478.23 (73.68)	433.69 (50.15)	0.718	0.002^‡^
Total sleep time	389.38 (61.93)	394.94 (67.05)	0.250	0.351^‡^
Sleep efficiency	82.05 (9.81)	90.99 (9.90)	0.880	0.000^†^

SD, Standard Deviation. ^†^Wilcoxon rank sum test, ^‡^dependent t-test.

We found large to very large improvements in sleep-related PROs, including sleep disturbance (*r* = 0.843, *p* = 0.000), sleep quality (*r* = 0851, *p* = 0.000), sleep hygiene practices (*r* = 0.770, *p* = 0.000), and a shift toward *morningness* in participants' chronotype preference (*r* = 0.713, *p* = 0.002; see Table 3). Mental health-related PROs improved including generalized anxiety (*r* = 0.503, *p* = 0.047) and depression (*r* = 0.600, *p* = 0.014). There were no adverse events.

**Table 3:** Change in outcomes from baseline to posttest and from posttest to 3-mo follow-up.

**Instruments**	**Baseline, mean (SD)**	**Posttest, mean (SD)**	**Follow-Up, mean (SD)**	**Baseline to posttest (*****n*** = **15)**		**Posttest to follow-Up (*****n*** = **10)**	
				**Effect size**, ***r***	***p*** **-value**	**Effect size**, ***r***	***p*** **-value**
**Sleep related outcomes**							
ISI-J	11.27 (2.60)	6.93 (4.48)	6.60 (2.63)	0.843	0.000^†^	0.037	0.915^‡^
PSQI-J	7.60 (3.33)	4.80 (2.37)	4.40 (1.43)	0.851	0.000^†^	0.217	0.521^‡^
J-ESS	11.60 (5.33)	7.87 (4.20)	6.60 (3.95)	0.614	0.011^‡^	0.413	0.207^‡^
DBAS-J	77.33 (18.07)	71.13 (19.55)	72.30 (20.89)	0.347	0.187^‡^	0.016	0.977^†^
SHPS-J	88.27 (21.54)	67.73 (15.18)	78.70 (26.70)	0.770	0.000^‡^	0.693	0.018^‡^
MEQ-J	41.20 (7.04)	46.13 (7.76)	46.60 (5.99)	0.713	0.002^‡^	0.383	0.245^‡^
**Mental health related outcomes**							
J-GAD-7	5.40 (4.27)	3.73 (2.99)	3.00 (2.62)	0.503	0.047^‡^	0.041	0.904^‡^
J-CESD	20.13 (9.16)	13.80 (7.61)	12.40 (5.91)	0.600	0.014^‡^	0.128	0.707^‡^
**Participation-related outcome**							
EMAS-J	35.67 (7.83)	37.40 (8.34)	39.20 (9.80)	0.258	0.334^‡^	0.030	0.930^‡^

ISI-J, Japanese version of the Insomnia Severity Index; PSQI-J, Japanese version of the Pittsburgh Sleep Quality Index; J-ESS, Japanese version of the Epworth Sleepiness Scale; DBAS-J, Japanese version of the Dysfunctional Beliefs and Attitudes about Sleep Scale; SHPS-J, Japanese version of the Sleep Hygiene Practice Scale; MEQ-J, Japanese version of the Morningness/Eveningness Questionnaire; J-GAD-7, Japanese version of the Generalized Anxiety Disorder-7; J-CESD, Japanese version of the Center for Epidemiologic Studies Depression Scale; EMAS-J, Japanese version of the Engagement in Meaningful Activities Survey; SD, Standard Deviation. ^†^Wilcoxon rank sum test, ^‡^Dependent t-test.

Comparing posttest (*n* = 15) and 3-mo follow up (*n* = 10) data we found most PRO effects were maintained. However, there was a large increase (*r* = 0.693. *p* = 0.018) in ineffective sleep behaviors (SHPS-J) at 3 mo follow-up and we explored these changes at the item level. Table 4 shows items from the SHPS-J with significant changes from pretest to posttest and posttest to 3-mo follow-up.

**Table 4:** Sleep hygiene practice scale items with significant change from baseline to posttest and/or from posttest to 3-mo follow-up.

**Item**	**Baseline, mean (SD)**	**Posttest, mean (SD)**	**Follow-Up, mean (SD)**	**Baseline to posttest**		**Posttest to follow-up**	
				**Effect size**, ***r***	***p*** **-value**	**Effect Size**, ***r***	***p*** **-value**
1. Bedtime not consistent daily.	4.27 (1.34)	3.00 (1.20)	4.10 (1.29)	0.631	0.010^†^	0.653	0.029^‡^
2. Get out of bed at inconsistent time.	4.07 (1.53)	3.00 (1.36)	4.10 (1.29)	0.509	0.044^‡^	0.707	0.015^‡^
3. Stay in bed after waking up in the morning.	4.60 (1.72)	2.73 (1.53)	3.90 (1.52)	0.656	0.008^†^	0.678	0.022^‡^
4. Sleep in on weekends.	5.07 (1.16)	3.00 (1.20)	4.80 (0.92)	0.749	0.002^†^	0.913	0.000^‡^
5. Doing sleep-irrelevant activities in bed (e.g., watching TV, reading).	5.20 (1.15)	2.60 (1.45)	4.20 (1.62)	0.785	0.001^†^	0.637	0.043^†^
6. Going to bed hungry	3.60 (1.35)	2.80 (1.32)	3.40 (1.43)	0.632	0.009^‡^	0.664	0.066^†^
7. Worry about not being able to fall asleep in bed.	3.07 (1.58)	1.87 (1.55)	1.90 (1.10)	0.658	0.010^†^	0.577	0.125^†^
11. Pondering about unresolved matters while lying in bed.	4.07 (1.94)	2.80 (1.47)	2.60 (1.78)	0.585	0.023^†^	0.141	0.678^‡^
12. Check the time in the middle of night.	4.20 (1.78)	3.40 (1.55)	3.00 (1.56)	0.560	0.039^†^	0.063	0.853^‡^
13. Napping or resting in bed for over one hour during the day.	3.53 (1.68)	2.40 (1.06)	2.60 (1.26)	0.703	0.002^‡^	0.169	1.000^†^
14. Lack of exposure to outdoor light during the day.	3.27 (1.67)	2.33 (1.29)	3.20 (1.48)	0.602	0.014^‡^	0.640	0.063^†^
21. Drinking a lot during the hour prior to sleep.	2.93 (1.53)	2.27 (1.28)	2.20 (1.48)	0.474	0.063^†^	0.298	0.531^†^
28. Uncomfortable bedding and/or pillow.	2.93 (1.67)	2.07 (1.10)	2.40 (1.43)	0.487	0.074^†^	0.696	0.031^†^

SD, Standard Deviation. ^†^Wilcoxon rank sum test, ^‡^Dependent t-test.

## 4 Discussion

Sleep Health through University Student Habits (SHUSH) was developed as a multi-component CBT-I intervention (Eakman et al., 2017) and adapted to the Japanese culture. We found the intervention improved sleep quality and mental health among undergraduate health sciences students, and these effects were sustained for 3 months. There were very large improvements from baseline to posttest in indicators of sleep health (Buysse, 2014) including reduced sleep disturbance (i.e., ISI-J) and improved sleep quality (i.e., PSQI-J). SHUSH was a brief intervention that began with a short one-hour group education session and included individually tailored sleep-behavior goals. We developed individualized sleep goals based upon five principles (see Table 1) for promoting effective sleep habits (e.g., wake up at the same time every day, get out of bed if not sleeping, limit napping). These behaviors can lessen insomnia symptom severity, promote sleep-wake synchrony and sleep quality (Perlis et al., 2005). On the other hand, the students did not report appreciable gains in total sleep time which remained at ~ 6.5 h on average. This finding is consistent with brief behavioral interventions for insomnia (Buysse et al., 2011) and indicate that additional weeks of therapy may be needed to improve overall sleep time (Muench et al., 2022).

### 4.1 Improvement in sleep-related and mental health-related outcomes

The improvements were seen on the sleep diary outcomes and sleep-related outcomes. The results were similar to those of the 6-week multicomponent CBT-I conducted with U.S. college students (Taylor et al., 2014). We believe students' understanding of sleep physiology provided by SHUSH, as well as personalized goal setting helped them value the intervention. Further, the weekly 1:1 face-to-face follow-up sessions between occupational therapists and students were likely helpful in promoting adherence to individualized sleep prescriptions (Lancee et al., 2016). SHUSH participants also showed improvements in mental health-related outcomes. This result was consistent with previous studies showing that CBT-I has a positive impact on the mental health of adults and Japanese university students (Ashworth et al., 2015; Ubara et al., 2022). Depression has been consistently associated with poor sleep in university samples (Dinis and Bragança, 2018; Becker et al., 2018) and worry, especially sleep-related worrying can contribute to the perpetuation of insomnia (Becker et al., 2018). Fortunately, a reduction in insomnia symptom severity can contribute to improved mental health following behavioral sleep interventions such as SHUSH.

### 4.2 Improvement in sleep habits among university students

The program is an intervention focused on the sleep habits of university students and, similar to previous studies, showed improvements in sleep hygiene behaviors. At posttest SHUSH was associated with significant improvements in sleep-promoting behaviors as assessed by the SHPS-J. We found item-level change for behaviors related to sleep timing and scheduling (SHPS-J items 1, 3, 4, 13 & 14). More often going to bed and getting out of bed at a consistent time, refraining from napping, and ensuring exposure to sunlight during activity were associated with improved sleep quality. We also found substantial improvements in behaviors contributing to cognitive arousal that can lessen sleepiness and extend sleep latency (SHPS-J items 5, 6, 11 & 12). As with general CBT-I, we encouraged participants to write down their thoughts and concerns before going to bed (Harvey and Farrell, 2003). Item 11, for example, regarding “pondering about unresolved matters while lying in bed” was improved. SHUSH may have helped reduce thoughts that lead to emotional arousal that may contribute to anxiety or depression. Students' most substantive posttest gain, however, was in item 5 reflecting engagement in fewer sleep-irrelevant activities in bed (e.g., watching TV, reading). The use of smartphones in bed is a major problem contributing to university students staying up later and waking later–likely delaying optimal circadian timing (Cui et al., 2021). Education content within SHUSH and personalized stimulus control goals, such as leaving the bed if not sleeping, likely contributed to positive behavior change (Perlis et al., 2005).

Perhaps occupational therapists' efforts to educate students on skills to manage worry and anxiety, and time management support to limit napping, may have contributed to these effects. We identified that students' lifestyles had varied demands (e.g., completing homework late into the night, being engaged with friends through the night, and part-time job demands for a late work schedule) which challenged adhering to sleep recommendations and provided obstacles for developing effective sleep habits. Effective time management will be important to future interventions, and use of smartphone applications to support sleep health behavior change may be useful (Thornton et al., 2021). Features such as self-monitoring tools, reminders, and prompts can be expected to enhance time management and sleep timing. Perhaps these tools, as well as face-to-face follow-up could help to support students in developing health promoting sleep routines.

### 4.3 Durability of SHUSH effects

The positive effects of SHUSH upon sleep disturbances and sleep quality persisted for 3 months after the program ended. Students continued to make gains, in fact, in the ISI-J and PSQI-J up to 3 mo after the SHUSH intervention. Daytime sleepiness saw a similar trend in improvement after 3 months. The MEQ-J gains were stable from posttest to 3 mo follow-up. This shift in circadian preference to a “morning type” may help explain the stability in SHUSH program effects upon sleep disturbance and sleep quality. Positive effects upon mental health were also sustained at 3-mo follow-up indicating modest durability for the intervention. The intervention was associated with improved sleep hygiene practices at posttest, though these effects were not sustained at 3 mo follow-up. We explored change at the item level and identified item 11 “worry or think in bed” and item 12 “check the time in the middle of the night” continued to improve after posttest. Item 13 related to napping behaviors was stable at 3 months indicating students continued to engage in less frequent napping. These sleep-related behaviors may have been useful in sustaining the SHUSH intervention effects upon sleep quality (Carney et al., 2009). Worry and sleep-related anxiety, especially in bed, may perpetuate insomnia by extending the time to fall asleep. Given that health science students have been shown to be the most affected in terms of insomnia and perceived stress (Busa et al., 2024), teaching them specific ways to manage stress could have a positive effect on their sleep. Napping, as well, may contribute to extended sleep latency and perpetuate insomnia, in part through delaying the sleep cycle and mitigating effective sleep regulation (Borbély et al., 2016).

We did observe a rebound effect for SHPS-J items 3, 4, 5, and 14 pretest to posttest, and posttest to 3-mo follow-up. Ineffective sleep-related behaviors that increased substantially at 3 mo follow-up included staying in bed after waking, sleeping in on weekends, sleep-irrelevant activities in bed, and a decrease in daylight-exposed activities. Increases in these four sleep-related activities could extend sleep onset latency and contribute to insomnia. We did not see this effect, however, and found that sleep quality and mental health gains were sustained at 3 mo follow-up despite a degradation in some sleep hygiene behaviors. Perhaps future studies could evaluate additional weekly follow-up meetings or a “booster” week to support students in modifying their sleep-related routines (Buysse, 2014). Students would benefit from ongoing professional support to embed sleep-promoting activities into their daily routines (Faulkner, 2022).

Cognitive and behavioral models of insomnia (Carney and Edinger, 2006) reflect that as ineffective sleep beliefs are modified through therapy, associated cognitions and behaviors may help drive improvements in sleep quality due to CBT-I. Students' reduced sleep-related worrying remained relatively low at follow-up which may suggest that sleep-related worrying and related cognitive arousal may be more amenable to change in a brief CBT-I intervention (Ballesio et al., 2021). Interestingly, students reported a rebound effect reflecting worsening sleep-related behaviors, yet these changes did not appear to impact their self-reported sleep quality or sleep problems at follow-up. These findings suggest that sleep-related worrying may have been more of a concern to poor sleep than sleep behaviors such as maintaining a consistent bed and rise times, sleeping in on weekends, as well as sleep-irrelevant activities in bed. As well, these findings may reflect that maintenance of behavioral changes may require a longer duration of therapy to ensure the formation of habitual behavioral changes to optimize the therapeutic effects of CBT-I (Muench et al., 2022).

### 4.4 Impact on participation-related outcomes

We did not find significant posttest change in the Engagement in Meaningful Activities Survey (EMAS-J), though we did see a trend in improvement across the three testing periods. We surmised that daytime difficulties in school life (i.e., absence, tardiness, falling asleep, academic performance) could be due in part to sleep disturbances (Kayaba et al., 2020). Further, we believed poor daytime performance could constrain the experience of meaningful activities. We noted recent evidence indicating occupational therapist-delivered CBT-I contributes to improved sleep health including lower daytime dysfunction and greater engagement in meaningful activities (i.e., EMAS). In the present study, we found a positive trend for the EMAS-J across the three testing periods (*Mean* [*SD*]: baseline = 35.67 [7.83], posttest = 37.40 [8.34] and 3-mo follow-up = 39.20 [3.10]), though this was not statistically significant. Given SHUSH was conducted in a short period of time it is likely the effects of improved sleep upon meaningful engagement had not been felt. Perhaps there is a necessary time lag to determine how improved sleep quality can improve participation. Future research could explore the timing and general influence of sleep quantity and quality upon meaningful engagement in day-to-day life.

### 4.5 Delivery by an occupational therapist

SHUSH was conducted by two occupational therapists with advanced training in multicomponent CBT-I. Given significant limitations in access to sleep health specialists, it is essential for occupational therapy to develop this area of practice. The SHUSH intervention utilized multicomponent CBT-I including stimulus control and sleep restriction therapies. These therapies are designed to encourage sleep-related behaviors that optimize sleep regulation and lessen the likelihood of perpetuating insomnia. Sleep intervention is becoming a prominent aspect of occupational therapy practice, especially as a means for promoting health (Green and Hicks, 2015; Carney et al., 2009). The professional should understand sleep physiology and methods to promote the synchrony of the circadian and sleep driver systems (Borbély et al., 2016). Specialty skill development in the safe and effective delivery of multicomponent CBT-I could aid the occupational therapy profession. Advanced knowledge will of course be required to inform occupational therapy practice and coordinate research across disciplines and areas of practice. Ultimately, sleep health promotion should be considered an area of specialization for rehabilitation providers such as occupational therapy (Tester and Foss, 2018).

## 5 Limitations

Unfortunately, 3rd grade students were doing fieldwork at the same time as this project. So, many students missed the opportunity to participate. The degree of overcrowding of the lecture schedule during this research period differed depending on the grade and department. It was difficult to control these conditions which may have influenced student availability to participate in SHUSH. Further, SHUSH was conducted under the COVID-19 pandemic. A more detailed analysis of the impact of demographic variables such as gender, grade and commute time are desirable in future studies. In addition, BMI, screen time, and exercise should be evaluated together in the future study. These studies require larger sample sizes. The results of this study may have been influenced by these factors. Collection of daily sleep diary data at 3 mo follow-up would improve understanding the effects of SHUSH. Lastly, causal assertions of SHUSH effectiveness are limited because this study lacked a control group and one third of the sample was lost to follow-up. Present findings, nonetheless, are consistent with a preponderance of evidence in support of multicomponent CBT-I as the first line treatment for insomnia (Qaseem et al., 2016).

## 6 Conclusions

SHUSH was developed as a brief cognitive-behavioral intervention for insomnia and it was delivered by occupational therapists with training in CBT-I. SHUSH is a feasible and preliminarily effective sleep improvement program for reducing sleep disturbances among Japanese university students. At 3 months follow-up sleep quality and mental health gains were maintained, though some sleep hygiene practices had regressed.

## Data Availability

The datasets presented in this article are not readily available because data cannot be shared publicly due to ethical restrictions imposed by the Yamagata Prefectural University Ethics Committee. Requests to access the datasets should be directed to Yuki Kawakatsu, ykawakatsu@yachts.ac.jp.
